# Effect of a short period of invasive mechanical ventilation following a successful spontaneous breathing trial in adults: a systematic review and meta-analysis

**DOI:** 10.62675/2965-2774.20260480

**Published:** 2026-07-07

**Authors:** Santiago Lucas Napoli, Aline Braz Pereira, Michelli Marcela Dadam, Alexandre Biasi Cavalcanti, João Gabriel Sanchez

**Affiliations:** 1 Hospital General de Agudos "Dr. Juan A. Fernández" Buenos Aires Argentina Hospital General de Agudos "Dr. Juan A. Fernández" - Buenos Aires, Argentina; 2 Centro Hospitalar Unimed Intensive Care Unit Joinville SC Brazil Intensive Care Unit, Centro Hospitalar Unimed - Joinville (SC), Brazil.; 3 Hospital Municipal São José Intensive Care Unit Joinville Santa Catarina Brazil Intensive Care Unit, Hospital Municipal São José - Joinville, Santa Catarina, Brazil; 4 HCor-Hospital do Coração Research Institute São Paulo SP Brazil Research Institute, HCor-Hospital do Coração - São Paulo (SP), Brazil.

**Keywords:** Ventilator weaning, Respiration, artificial, Airway extubation, Rest, Reintubation

## Abstract

**Objective:**

We aimed to assess the effect of a short period of invasive mechanical ventilation after a successful spontaneous breathing trial, compared with immediate extubation, on the risk of reintubation within 48 hours.

**Methods:**

We conducted a systematic review and meta-analysis of randomized clinical trials comparing a short period of invasive mechanical ventilation following a successful spontaneous breathing trial versus immediate extubation. We searched PubMed^®^, Cochrane Central, Embase, Scopus, and Web of Science. Pairs of reviewers independently screened studies, extracted data, and assessed risk of bias with the Cochrane Risk of Bias tool 2.0. The primary outcome was reintubation within 48 hours after randomization. Data were pooled using a Hartung-Knapp-Sidik-Jonkman random-effects model. Trial sequential analysis was performed to determine whether the accumulated evidence was sufficient for definitive conclusions. Certainty of evidence was assessed using the GRADE approach. Secondary outcomes were reintubation or death within 7 days after extubation, intensive care unit and hospital length of stay, in-hospital mortality, and ventilator-free days up to day 28.

**Results:**

Our search identified 1,473 unique records, of which 4 randomized clinical trials (n = 1,071 patients) were eligible. The pooled risk ratio for reintubation within 48 hours in patients receiving a short period of invasive mechanical ventilation after a successful spontaneous breathing trial, compared with immediate extubation, was 0.48 (95% confidence interval 0.22 - 1.07; p = 0.06; I^2^ = 41.6%). Trial sequential analysis confirmed that the current evidence base is underpowered for definitive conclusions. The evidence was rated as low due to serious inconsistency and imprecision. No significant differences were observed for any of the secondary outcomes.

**Conclusions:**

Among critically ill adults, a short period of invasive mechanical ventilation after a successful spontaneous breathing trial did not significantly reduce the risk of extubation failure within 48 hours compared with immediate extubation (low-certainty evidence). Further adequately powered trials are needed to clarify the clinical efficacy of this intervention.

## INTRODUCTION

Mechanical ventilation (MV) remains a cornerstone of treatment for critically ill patients, providing vital support for conditions such as acute respiratory failure, hemodynamic instability, airway compromise, and other critical illnesses. However, prolonged MV is associated with ventilator-induced lung injury, ventilator-associated pneumonia, *delirium*, muscle atrophy, prolonged intensive care unit (ICU) and hospital stays, increasing morbidity and mortality.^(1)^

Healthcare professionals implement strategies to mitigate these risks by minimizing the duration of MV and reducing the likelihood of extubation failure. Among these approaches, spontaneous breathing trials (SBT) are an important part of weaning protocols, assessing a patient's ability to breathe independently and predicting extubation failure.^(2-8)^ Most utilized techniques for SBT are T-piece trials and pressure support ventilation. Nevertheless, the optimal method remains a topic of ongoing debate, as evidence remains inconsistent.^(9,10)^

A short period of invasive MV following a successful SBT has been proposed to reduce the risk of extubation failure.^(11-15)^ This brief interval of ventilatory support may allow the cardiovascular system to return to pre-SBT status and the respiratory muscles to rest. In addition, a physiological study showed that SBTs – particularly those performed with a T-piece – are associated with a significant decrease in end-expiratory lung volume, and that providing one hour of MV after a successful SBT led to full recovery of end-expiratory lung volume, regardless of the SBT modality.^(13)^ However, this strategy may delay extubation, require reinitiation of sedation, and compromise optimal timing for extubation. At least two randomized clinical trials (RCTs) assessed one hour of MV following a successful SBT in a T-piece. Fernandez et al. reported a reduction in the risk of reintubation within 48 hours after randomization,^(11)^ whereas Dadam et al. found no significant difference in this outcome.^(12)^ Additionally, two smaller trials have reported inconsistent findings.^(14,15)^

To date, no systematic review or meta-analysis has addressed the clinical efficacy of this practice. Therefore, we conducted a systematic review and meta-analysis to assess the effect of a short period of invasive MV following a successful SBT, compared with immediate extubation, on the risk of reintubation within 48 hours.

## METHODS

We registered and published an online protocol for this systematic review on the International Prospective Register of Systematic Reviews (PROSPERO, registration number CRD42025636101; January 21, 2025). Institutional review board approval and informed consent were not required, as this study involved secondary analysis of previously published data. The review was conducted in accordance with the Cochrane Handbook for Systematic Reviews of Interventions and is reported following the Preferred Reporting Items for Systematic Reviews and Meta-Analyses (PRISMA 2020) guidelines.^(16,17)^

### Data sources and search strategy

We conducted a systematic literature search without restriction on language or publication date, from database inception to December 23, 2024. The following electronic databases were searched: PubMed^®^ (MEDLINE^®^), Cochrane CENTRAL, Embase, Scopus, and Web of Science. In addition, we searched the reference lists of included studies and gray literature sources, trial registries, and Google Scholar to identify additional relevant studies. The complete list of databases and detailed search strategies is provided in table 1S (Supplementary Material).

### Eligibility criteria

We included parallel-group RCTs enrolling adult patients (≥ 18 years) admitted to ICUs, receiving invasive MV via endotracheal tube, who had successfully completed an SBT and were planned for extubation. Eligible studies compared a short period of invasive MV following a successful SBT, defined as a return to MV with previous ventilatory settings for a short period (range 20 minutes to 180 minutes) before extubation, with immediate extubation. Included studies must report at least one of the following outcomes: reintubation within 48 hours from randomization, or weaning failure, defined as reintubation or death within 7 days of extubation, in accordance with the WIND classification.^(18)^

We excluded observational studies, case reports, case series, reviews and opinion articles, animal studies, studies conducted outside ICU settings, those involving pediatric patients, trials enrolling patients who were not intended for extubation despite a successful SBT, or studies involving patients receiving MV using other strategies which do not involve an endotracheal tube.

### Study selection and data extraction

Two independent, blinded reviewers performed study selection and data extraction. A third reviewer adjudicated disagreements and established consensus within the study team. The process was carried out in two phases using the Rayyan tool.^(19)^ The first phase was the selection of studies by reading the titles and abstracts of the articles identified in the search. The second phase involved reading the studies included in the first phase in full. For the data extraction, we use a previously prepared, tested, and validated spreadsheet to retrieve relevant information, including primary and secondary outcomes, and subgroup analysis data.

The primary outcome was reintubation within 48 hours of randomization. Secondary outcomes were reintubation or death within 7 days after extubation (weaning failure, as defined by the WIND classification),^(18)^ ICU length of stay, hospital length of stay, in-hospital mortality, and ventilator-free days up to day 28.

### Risk of bias and quality assessment

Two reviewers independently assessed risk of bias using the Cochrane Risk of Bias 2.0 instrument.^(20,21)^ The following domains were evaluated: random sequence generation, allocation concealment, blinding of participants and personnel, blinding of outcome assessment, incomplete outcome data, selective outcome reporting, and other biases (e.g., imbalanced baseline characteristics, inadequate funding, etc.). We performed a separate risk-of-bias assessment for each outcome of interest and classified the overall risk of bias for each outcome as low, some concerns, or high. In cases of missing or unclear data on study characteristics, risk of bias, or outcomes, we contacted the study authors directly. Disagreements between reviewers were resolved through discussion or, when necessary, adjudicated by a third reviewer. We assessed the certainty of evidence using the Grading of Recommendations Assessment, Development and Evaluation (GRADE) approach for each important outcome and presented the results accordingly.^(22)^

### Statistical analysis

We reported the primary outcome as a dichotomous variable, with relative risk (RR) and corresponding 95% confidence intervals (95%CI) as the measure of effect. For secondary outcomes, we analyzed dichotomous variables using RR with 95%CI and continuous variables using the mean difference (MD) with 95%CI. We pooled results using a random-effects meta-analysis.

Given the small number of included studies and their relatively small sample sizes, we used the Hartung-Knapp-Sidik-Jonkman method for all primary analyses. We conducted sensitivity analyses using the DerSimonian-Laird method to assess whether the ad hoc variance correction is appropriate. When the confidence interval obtained with the Hartung-Knapp-Sidik-Jonkman method was narrower than that with the DerSimonian-Laird method, we applied the Hartung-Knapp-Sidik-Jonkman method with an ad hoc variance correction. This approach adjusts for uncertainty in between-study variance estimation, providing more robust, conservative confidence intervals under such conditions.^(23-30)^

We tested the overall effect size using a t statistic, as implemented in the Hartung-Knapp-Sidik-Jonkman method, to determine whether the pooled effect estimate significantly differed from the null, while accounting for uncertainty in the estimation of between-study variance. We assessed statistical heterogeneity among the included studies quantitatively using the Q test and I^2^ statistic, and qualitatively through visual inspection of forest plots to evaluate the degree of overlap in CIs.

### Trial sequential analysis

We performed trial sequential analysis (TSA) for the primary outcome to assess the conclusiveness of the pooled evidence and to reduce the risk of type I error arising from sparse data and repeated significance testing. We conducted TSA using a random-effects model with variance adjustment via the Hartung-Knapp-Sidik-Jonkman method. We constructed sequential monitoring boundaries for benefit and futility based on the O’Brien-Fleming alpha-spending function. To correct for heterogeneity, we applied model variance derived from the I^2^ statistic obtained from the random-effects meta-analysis. We calculated the required information size using a two-sided α of 0.05, a β of 0.10, an anticipated RR reduction of 20% with one hour of MV intervention, and a control event proportion of 24% based on previous studies.^(12,14)^ We plotted the cumulative Z-curve to evaluate the strength of the evidence. Both conventional boundaries and trial sequential monitoring boundaries were established for the two groups. When the Z-curve crosses the trial sequential monitoring boundary or enters the futility zone, the available evidence is considered conclusive, and additional trials are unnecessary. Conversely, if the Z-curve does not cross any boundary, the evidence remains inconclusive, indicating that further studies are required. We performed all TSA analyses using Trial Sequential Analysis software version 0.9.5.10 Beta.^(31)^

### Subgroup analysis

For the primary outcome, we prespecified and performed subgroup analyses based on the following criteria and expected directions of effect: (1) type of SBT: T-piece or pressure support ventilation; (2) duration of the short period of invasive MV following a successful SBT: trials with MV < 60 minutes, trials with MV of exactly 60 minutes, trials with MV > 60 minutes; (3) risk of bias: low risk of bias, high risk of bias; (4) risk of extubation failure: low risk of extubation failure, high risk of extubation failure; (5) duration of previous MV: up to 72 hours of MV, 72 hours or more of MV. Analyses were performed based on the data available in the included studies.

## RESULTS

### Study selection and characteristics

**Figure 1:**
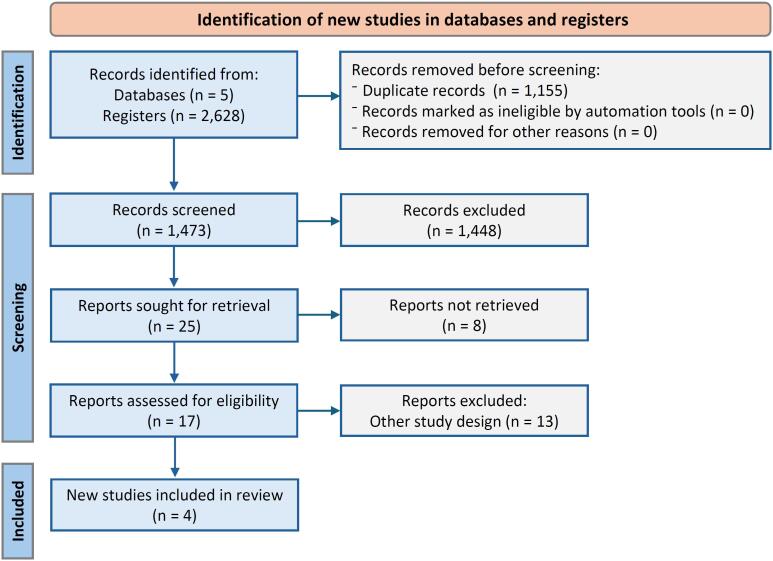
Flow diagram of study screening and selection based on Preferred Reporting Items for Systematic Reviews and Meta-Analysis.

We identified a total of 2,628 results from the following databases: PubMed^®^ (n = 433), Embase (n = 667), Cochrane Library (n = 510), Scopus (n = 642), and Web of Science (n = 376). After removing 1,155 duplicates, we screened 1,473 titles and abstracts using the Rayyan platform. The screening excluded 1,448 publications, and 25 full-text articles were assessed for eligibility. Finally, four publications met the inclusion criteria and were included in the analysis (Figure 1). The included RCTs were published between 2017 and 2025. Two studies were conducted in Brazil,^(12,14)^ one in Saudi Arabia,^(15)^ and one in Spain.^(11)^ A total of 1,071 patients were included, with 530 assigned to the intervention group and 541 to the control group. In all studies, the short invasive MV period following a successful SBT lasted 1 hour. An SBT was conducted using a T-piece in 798 (74.5%) patients, pressure support in 73 (6.8%) patients, and the method was not reported in 300 patients (28%). Most patients met criteria for high risk of extubation failure. Additional study characteristics are summarized in table 1 and table 2S (Supplementary Material).

**Table 1:** Characteristics of included studies

Study	Design	Country	Sites (number)	Setting	Intervention	Control	Sample	Duration of previous MV	Type of SBT
Fernandez et al.^(11)^	RCT	Spain	17	Medical/surgical ICUs	One hour of MV after a successful SBT, followed by extubation	Immediate extubation	470 patients	More than 12 hours	T-piece (90.9%) PS or CPAP (9.1%)
Dadam et al.^(12)^	RCT	Brazil	3	Medical/surgical ICUs	One hour of MV after a successful SBT, followed by extubation	Immediate extubation	336 patients	More than 12 hours	T piece (100%)
Pereira et al.^(14)^	RCT	Brazil	4	Medical/surgical ICUs	One hour of MV after a successful SBT, followed by extubation	Immediate extubation	65 patients	More than 72 hours	T piece (54%) PSV (46%)
Allam^(15)^	RCT	Saudi Arabia	1	Not specified	One hour of MV before extubation	Immediate extubation	300 patients	More than 7 days	Not specified

SBT - spontaneous breathing trial; RCT - randomized clinical trial; ICU- intensive care unit; MV - mechanical ventilation; PS - pressure support; CPAP - continuous positive airway pressure.

### Pooled analysis of all outcomes

#### Primary outcome

**Figure 2:**
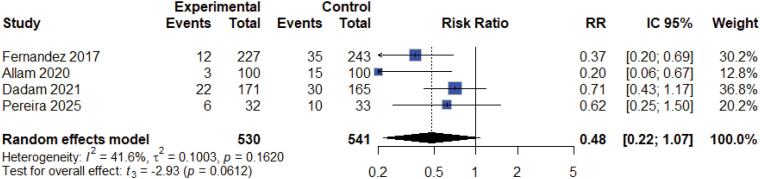
Pooled risk ratio of reintubation within 48 hours of randomization in patients treated with 1 hour of mechanical ventilation (experimental group) and immediate extubation (control group), using the Hartung-Knapp-Sidik-Jonkman method.

Reintubation within 48 hours of randomization was reported in three studies. The trial by Allam^(15)^ did not report reintubation within 48 hours but did report it within 24 hours. We contacted the authors to request the 48-hour data, but did not receive a response. Therefore, we used the 24-hour reintubation data from Allam^(15)^ in the primary analysis (Figure 2). The meta-analysis, including all four trials, yielded a pooled risk ratio of 0.48 (95%CI 0.22 - 1.07; p = 0.06; I^2^ = 41.6%) for 1-hour MV *versus* immediate extubation. A sensitivity analysis using the DerSimonian-Laird method demonstrated a statistically significant benefit in favor of the intervention (pooled risk ratio 0.48, 95%CI 0.30 - 0.78; p = 0.003, I^2^ = 41.6%) (Figure 1S - Supplementary Material).

**Figure 3:**
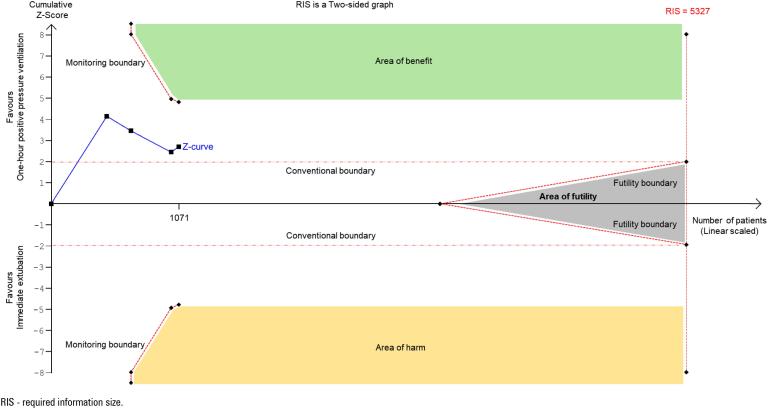
Trial sequential analysis for reintubation within 48 hours.

According to the TSA (Figure 3), the cumulative Z-curve neither crossed the efficacy, safety, or futility boundaries, nor attained the required information size, thereby indicating insufficient evidence and inconclusive findings. The diversity-adjusted required information size was calculated as 5,327 participants, compared with the 1,071 participants accrued across the included RCTs.

The cumulative Z-curve neither crossed the futility boundary nor reached the required information size, suggesting insufficient evidence and inconclusive results. A diversity-adjusted required information size of 5,327 patients was calculated using a two-sided α of 0.05, a β of 0.10, an anticipated relative risk reduction of 20% with the intervention, and a control event proportion of 24% based on previous studies^(12,14)^ and a heterogeneity correction using model variance based on the I^2^ statistic equal to 42%.

#### Secondary outcomes

For the secondary outcomes, we included data from three of the four studies for weaning failure defined by the WIND criteria (Figures 2S and 3S - Supplementary Material), ICU length of stay (Figures 4S and 5S - Supplementary Material), and hospital length of stay (Figures 6S and 7S - Supplementary Material). No statistically significant differences were found between groups.

Only two studies reported mortality data.^(12,14)^ The pooled analysis suggested a potential increase in mortality associated with one-hour MV (Figure 8S - Supplementary Material). However, post-hoc sensitivity analyses using the DerSimonian-Laird method and the modified Hartung-Knapp adjustment with ad hoc variance correction did not show a statistically significant effect (Figures 9S and 10S - Supplementary Material). Finally, data on 28-day ventilator-free days were not available in most of the included studies.

### Subgroup analysis

We conducted subgroup analysis based on baseline risk of extubation failure, as shown in figures 11S and 12S (Supplementary Material). In studies assessed as having low risk of bias, the pooled risk ratio for reintubation within 48 hours favored the intervention but did not reach statistical significance (RR 0.55; 95%CI 0.22 - 1.36; p = 0.10, I^2^ = 22.9%) (Figure 13S - Supplementary Material). We did not perform subgroup analyses by type of spontaneous breathing trial or by duration of previous MV, as most studies did not report sufficient data for these variables. Similarly, subgroup analysis by MV duration after the SBT was not conducted, as all included trials used the same 60-minute duration.

### Risk of bias

We classified one study as high risk of bias due to concerns about the randomization process and selective outcome reporting.^(15)^ The remaining studies were classified as having a low risk of bias across all assessed domains (Table 2).^(11,12,14)^

**Table 2:** Risk of bias

Study	Analysis	Experimental	Comparator	Primary outcome	Weight	D1	D2	D3	D4	D5	Overall
Fernandez et al.^(11)^	ITT	One hour of MV after a successful SBT	Immediate extubation	Reintubation within 48 hours	NA						
Dadam et al.^(12)^	ITT	One hour of MV after a successful SBT	Immediate extubation	Reintubation within 48 hours	NA						
Pereira et al.^(14)^	ITT	One hour of MV after a successful SBT	Immediate extubation	Extubation failure within 7 days	NA						
Allam^(15)^	ITT	One hour of MV after a successful SBT	Immediate extubation	Reintubation within 48 hours	NA	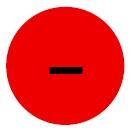				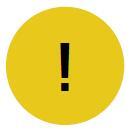	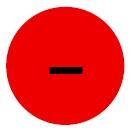

D1 - randomization process; D2 - deviations from the intended interventions; D3 - missing outcome data; D4 - measurement of the outcome; D5 - selection of the reported result; ITT - intention-to-treat; MV - mechanical ventilation; SBT - spontaneous breathing trial; NA - not available. 

 low risk; 
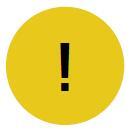
 some concerns; 
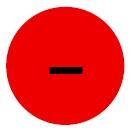
 high risk.

### Quality assessment

We assessed the certainty of evidence using the GRADE approach, and the results are presented in table 3. For the primary outcome (reintubation within 48 hours), the certainty of evidence was rated as low due to serious inconsistency and imprecision. For secondary outcomes (ICU and hospital length of stay, mortality, and weaning failure), the certainty of evidence ranged from low to very low, primarily due to concerns related to inconsistency, imprecision, and publication bias across the included trials.

**Table 3:** Summary of findings

Outcomes	Anticipated absolute effects* (95%CI)		Relative effect (95%CI)	Number of participants (studies)	Certainty of the evidence (GRADE)	Comments
	Risk with immediate extubation	Risk with short-period MV after a successful spontaneous breathing trial				
Reintubation within 48 hours of randomization	166 per 1.000	80 per 1.000 (37 to 178)	RR 0.48 (0.22 to 1.07)	1.071 (4 RCTs)	⨁⨁◯◯ Low† ‡	Low due to serious inconsistency and imprecision
Reintubation or death within 7 days after extubation according to the WIND classification	211 per 1.000	143 per 1.000 (46 to 456)	RR 0.68 (0.22 to 2.16)	871 (3 RCTs)	⨁◯◯◯ Very low† §	Very low due to serious inconsistency and very serious imprecision
ICU length of stay	The mean ICU length of stay was 0 SD	MD 0.17 SD lower (1.67 lower to 1.34 higher)	-	871 (3 RCTs)	⨁⨁⨁◯ Moderate¶	Moderate due to serious imprecision
Hospital length of stay	The mean hospital length of stay was 0 SD	MD 2.24 SD higher (4.1 lower to 8.58 higher)	-	871 (3 RCTs)	⨁⨁◯◯ Low§	Low due to very serious imprecision
In-hospital mortality	162 per 1.000	202 per 1,000 (192 to 212)	RR 1.25 (1.19 to 1.31)	401 (2 RCTs)	⨁◯◯◯ Very low§ ||	Very low due to very serious imprecision and serious risk of publication bias

* The risk in the experimental group (and its 95% confidence interval) is based on the assumed risk in the comparison group and the relative effect of the intervention (and its 95%CI);

† serious inconsistency;

‡ imprecision

¶ serious imprecision

§ very serious imprecision

|| serious risk of publication bias. 95%CI - 95%confidence interval; MV – mechanical ventilation; RR - risk ratio; RCT - randomized clinical trials; ICU – intensive care unit; SD - standard deviation; MD - mean difference.

GRADE Working Group grades of evidence. High certainty: we are very confident that the true effect lies close to that of the estimate of the effect. Moderate certainty: we are moderately confident in the effect estimate - the true effect is likely to be close to the estimate of the effect, but there is a possibility that it is substantially different. Low certainty: our confidence in the effect estimate is limited: the true effect may be substantially different from the estimate of the effect. Very low certainty: we have very little confidence in the effect estimate - the true effect is likely to be substantially different from the estimated effect.

## DISCUSSION

This systematic review and meta-analysis included 4 RCTs enrolling 1,071 critically ill patients who were randomized to a short period of invasive MV after a successful SBT or to immediate extubation. The intervention did not significantly decrease the risk of reintubation within 48 hours, with low certainty of evidence due to serious inconsistency and imprecision. No statistically significant differences were observed in secondary outcomes, including ICU and hospital length of stay, weaning failure according to the WIND classification (reintubation or death within 7 days), or mortality.

Strengths of this systematic review include a comprehensive literature search and detailed evaluation of the effects of a short period of invasive MV following a successful SBT. This review also highlights the limited data available on this strategy, particularly following SBT conducted with pressure support, and underscores the need for further research in both pressure-support and T-piece SBT populations.

This review has limitations. First, the small number of studies made it challenging to perform sensitivity analyses to assess heterogeneity. Second, there was substantial clinical variability across trials, including differences in the duration of MV prior to extubation and in patients’ baseline risk of extubation failure. These variations, along with high unexplained heterogeneity and imprecision, directly affected the overall certainty of the evidence. One of the included RCTs reported extubation failure only within 24 hours, whereas the other studies used a 48-hour window. No additional data were obtained despite attempts to contact the author. This discrepancy in outcome definition may lead to underestimation of failure events in this study, introducing heterogeneity and potentially biasing pooled estimates. Sensitivity analyses were performed to assess the impact of including this study. Third, although the Hartung-Knapp-Sidik-Jonkman method for random-effects meta-analysis generally provides more accurate error rates than the conventional DerSimonian-Laird method, particularly when the number of studies is small,^(23)^ caution is warranted when interpreting results based on very few studies, especially when sample sizes are highly unbalanced and heterogeneity is very low. In such scenarios, Hartung-Knapp-Sidik-Jonkman confidence intervals may become overly conservative or deceptively narrow, potentially leading to misleading inferences if interpreted at face value.^(24,25)^ To address this in the mortality meta-analysis, we applied a modified Hartung-Knapp-Sidik-Jonkman approach with an ad hoc variance correction.^(26,27)^ However, because the analysis included only two studies, this correction may remain overly conservative, and the issue remains statistically unresolved.^(23-30)^

The pooled point estimate suggested a 52% RR reduction in reintubation within 48 hours with the intervention. However, the estimate was accompanied by substantial imprecision, with 95%CIs compatible with either a large benefit or a small harmful effect. Trial sequential analysis confirmed that the current sample size remains insufficient to support definitive conclusions. Moderate heterogeneity was observed, and subgroup analyses failed to identify its sources. Notably, most included patients underwent a T-piece SBT, limiting generalizability to those extubated after pressure support SBT.

A short period of invasive MV following a successful SBT is a low-cost, low-complexity intervention with minimal risk of complications. The mechanism by which this intervention may reduce the risk of extubation failure remains incompletely understood. A physiological study suggests that SBT may lead to alveolar derecruitment, which can be fully reversed by a brief period of post-trial ventilatory support.^(13)^ Dadam et al. demonstrated that this intervention may reduce extubation failure in patients mechanically ventilated for more than 72 hours.^(12)^ Prolonged MV is known to be associated with complications such as ICU-acquired weakness^(32-34)^ and diaphragmatic dysfunction,^(35-37)^ both of which may contribute to extubation failure. In a randomized trial, Thille et al.^(9)^ compared SBT with pressure support *versus* a T-piece in high-risk patients, reporting a reintubation rate of 15%, lower than typically observed in this population.^(38-40)^ This may be partly explained by the use of a short period of invasive MV following a successful SBT in both intervention groups, as well as prophylactic noninvasive ventilation after extubation in approximately 80% of patients.^(9)^ Given the multifactorial nature of weaning management, these interventions likely exert complementary effects that enhance overall clinical benefit.

Future large, well-designed trials are needed to clarify whether this intervention reduces the risk of extubation failure. Clarifying this question is clinically relevant, as extubation failure is associated with an increased risk of ventilator-associated pneumonia, prolonged hospital stay, higher mortality, and greater healthcare costs.^(1)^ Ultimately, such trials could meaningfully improve outcomes for critically ill patients and enhance the quality of care in ICUs.

## CONCLUSION

Among critically ill patients who have successfully completed a spontaneous breathing trial, a short period of invasive mechanical ventilation compared with immediate extubation has inconclusive effects on reintubation within 48 hours (low-certainty evidence) and other clinical outcomes. Accordingly, adequately powered randomized clinical trials are needed to address this question in both populations: those who pass a T-piece trial and those who complete a pressure support spontaneous breathing trial.

## Data Availability

The contents will be made available at the time of publication of the article. Contents underlying the article text: 10.5281/zenodo.20263242
