# Supplementary material for: Effect of a short period of invasive mechanical ventilation following a successful spontaneous breathing trial in adults: a systematic review and meta-analysis
Source: Crit Care Sci. 2026 Jul 7;38:e20260480. doi: 10.62675/2965-2774.20260480 (PMC13399242; doi:10.62675/2965-2774.20260480)
Supplement: Supplementary Material [file 2965-2774-ccsci-38-e20260480-Suppl01.pdf]

*Critical Care Science*
https://doi.org/10.62675/2965-2774.20260480

SUPPLEMENTARY MATERIAL

# Effect of a short period of invasive mechanical ventilation following a successful spontaneous breathing trial in adults: a systematic review and meta-analysis

Santiago Lucas Napoli[1], Aline Braz Pereira[2], Michelli Marcela Dadam[3], Alexandre Biasi Cavalcanti[4], João Gabriel Sanchez[4]

**Table 1S -** Databases and search strategy

| Database | Search strategy | Results |
|---|---|---|
| PubMed® | (((((spontaneous breathing trial[Title/Abstract]) OR (SBT[Title/Abstract])) OR (T-piece[Title/Abstract])) OR ((((Critical Illness[Title/Abstract]) OR (critically ill[Title/Abstract])) OR (Critical Illnesses[Title/Abstract])) OR (((“Intensive Care Units”[Mesh]) OR “Critical Illness”[Mesh]) OR ((Intensive Care Unit*[Title/Abstract]) OR (ICU[Title/Abstract]))))) AND (((Spontaneous Breathing[Title/Abstract]) OR (reconnection to mechanical ventilation[Title/Abstract])) OR (((“Respiration, Artificial”[Mesh]) OR ((Mechanical ventilatory support[Title/Abstract]) OR (mechanical ventilation[Title/Abstract]))) OR (mechanically ventilated[Title/Abstract])))) AND (((reintubation[Title/Abstract]) OR (Weaning failure[Title/Abstract])) OR (Extubation failure[Title/Abstract]))) AND ((((((randomized controlled trial[Publication Type]) OR (controlled clinical trial[Publication Type])) OR (randomized[Title/Abstract])) OR (clinical trials as topic[MeSH Major Topic])) OR (randomly[Title/Abstract])) OR (trial[Title])) | 433 |
| Embase | (((((‘spontaneous breathing trial’:ti,ab,kw) OR (‘sbt’:ti,ab,kw)) OR (‘t-piece’:ti,ab,kw)) OR ((((‘critical illness’:ti,ab,kw) OR (‘critically ill’:ti,ab,kw)) OR (‘critical illnesses’:ti,ab,kw)) OR (((‘intensive care unit’/exp) OR ‘critical illness’/exp) OR ((‘intensive care unit*’:ti,ab,kw) OR (‘icu’:ti,ab,kw))))) AND (((‘spontaneous breathing’:ti,ab,kw) OR (‘reconnection to mechanical ventilation’:ti,ab,kw)) OR (((‘artificial ventilation’/exp) OR ((‘mechanical ventilatory support’:ti,ab,kw) OR (‘mechanical ventilation’:ti,ab,kw))) OR (‘mechanically ventilated’:ti,ab,kw)))) AND (((‘reintubation’:ti,ab,kw) OR (‘weaning failure’:ti,ab,kw)) OR (‘extubation failure’:ti,ab,kw))) AND ((((((‘randomized controlled trial’:it) OR (‘controlled clinical trial’:it)) OR (‘randomized’:ti,ab,kw)) OR (‘clinical trial (topic)’/exp/mj)) OR (‘randomly’:ti,ab,kw)) OR (‘trial’:ti)) | 667 |
| Cochrane Library (trials) | Search Name:<br>Date Run: 23/12/2024 19:31:52<br>Comment:<br>ID Search Hits<br>#1 (“spontaneous breathing trial” OR “SBT” OR “T-piece” OR “Critical Illness” OR “critically ill” OR “Critical Illnesses” OR “Intensive Care Unit” OR “ICU”):ti,ab,kw 39963<br>#2 (“Spontaneous Breathing” OR “reconnection to mechanical ventilation” OR “Mechanical ventilatory support” OR “mechanical ventilation” OR “mechanically ventilated”):ti,ab,kw 17618<br>#3 (“reintubation” OR “Weaning failure” OR “Extubation failure”):ti,ab,kw 1394<br>#4 (“randomized controlled trial” OR “controlled clinical trial” OR “randomized” OR “randomly” OR “trial”):ti,ab,kw 1504804<br>#5 #1 AND #2 AND #3 AND #4 | 510 |
| Scopus | ( TITLE-ABS-KEY ( “spontaneous breathing trial” OR sbt OR “T-piece” OR “critically ill” OR “Critical Illness” OR “Critical Ill” ) AND TITLE-ABS-KEY ( “Spontaneous Breathing” OR “reconnection to mechanical ventilation” OR “Mechanical ventilatory support” OR “mechanical ventilation” OR “mechanically ventilated” ) AND TITLE-ABS-KEY ( “randomized controlled trial” OR “controlled clinical trial” OR “randomized” OR “trial” ) AND TITLE-ABS-KEY ( reintubation OR reconnection OR “Weaning failure” OR “Extubation failure” ) ) | 642 |
| Web of Science | ((((((TI=(Spontaneous Breathing)) OR TI=(reconnection to mechanical ventilation)) OR TI=(Mechanical ventilatory support)) OR TI=(mechanical ventilation)) OR TI=(mechanically ventilated)) OR TI=(reintubation)) OR TI=(Extubation)) AND (((TI=(spontaneous breathing trial)) OR TI=(SBT)) OR TI=(T-piece)) | 376 |
| Total | | 2,628 |
| Duplicates | | 1,155 |
| Total without duplicates (screening) | | 1,473 |

Date access: 23.12.2024



This is an open access article under the CC BY license (https://creativecommons.org/licenses/by/4.0/).

**Table 2S -** Data extraction

| Study | Fernandez et al.[1] | Allam[2] | Dadam et al.[3] | Pereira et al.[4] |
|---|---|---|---|---|
| Participants, n | EG: 227<br>CG: 243 | EG: 100<br>CG: 100 | EG: 171<br>CG: 165 | EG: 32<br>CG: 33 |
| Primary outcome | | | | |
| RR of reintubation within 2 days from randomization | 0.36 (95%CI 0.19 - 0.68) | 0.30 (95%CI 0.17 - 0.52) | 0.71 (95%CI 0.43 - 1.18) | 0.61 (95%CI 0.25 - 1.50) |
| Secondary outcomes | | | | |
| Weaning failure* | Not available data | Not available data | 1.03 (95%CI 0.71 - 1.49) | 0.66 (95%CI 0.33 - 1.31) |
| ICU length of stay (days) | EG: 11 (6 - 18)<br>CG: 10 (5 - 19) | Not available data | EG: 10 (6.5 - 15)<br>CG: 10 (5 - 18) | EG: 12.5 (6 - 17.5)<br>CG: 11 (8 - 20) |
| Hospital length of stay (days) | EG: 26 (17 - 43)<br>CG: 23 (14 - 38) | Not available data | EG: 22 (14 - 36)<br>CG: 23 (13 - 36) | EG: 26.5 (17.7 - 53.5)<br>CG: 24 (16 - 39) |
| In-hospital mortality | Not available data | Not available data | EG: 35 (20.3%)<br>CG: 27 (16.3%)<br>RR 1.25<br>(95%CI 0.79 - 1.96) | EG: 6 (19%)<br>CG: 5 (15%)<br>RR 1.24<br>(95%CI 0.42 - 3.63) |
| Ventilator-free days at day 28 | Not available data | Not available data | Not available data | IG: 28 (23 - 28)<br>CG: 28 (17 - 28) |
| Duration of MV after SBT | 60 minutes | 60 minutes | 60 minutes | 60 minutes |
| High risk of extubation failure | EG: 190 (84%)<br>CG: 202 (85%) | 100% | EG: 118 (69%)<br>CG: 115 (69.7%) | EG: 31 (96.8%)<br>CG: 31 (94%) |
| Duration > 72 hours of previous MV | Not available data | 100% | EG 115 (67.3%)<br>CG: 118 (71.5%) | 100% |

EG- Experimental Group; CG - Control Group; RR - relative risk; 95%CI - 95% confidence interval; ICU - intensive care unit; MV - mechanical ventilation; SBT - spontaneous breathing trial; PS - pressure support; CPAP - continuous positive airway pressure. * Weaning failure according to WIND definition: reintubation or death within 7 days after extubation.

## REFERENCES


1. Fernandez MM, González-Castro A, Magret M, Bouza MT, Ibañez M, García C, et al. Reconnection to mechanical ventilation for 1 h after a successful spontaneous breathing trial reduces reintubation in critically ill patients: a multicenter randomized controlled trial. Intensive Care Med. 2017;43(11):1660-7.
2. Allam MG. Use of either non-invasive ventilation immediately post-extubation or controlled mechanical ventilation for one hour after fulfilling weaning criteria decreases re intubation of patients with post-traumatic ARDS. Open Anesth J. 2020;15(1):7-19.
3. Dadam MM, Gonçalves AR, Mortari GL, Klamt AP, Hippler A, Lago JU, et al. The effect of reconnection to mechanical ventilation for 1 hour after spontaneous breathing trial on reintubation among patients ventilated for more than 12 hours: a randomized clinical trial. Chest. 2021;160(1):148-56.
4. Pereira A, Dadam MM, Catelano BA, Delvan D, Pastorello VH, Radun LC, et al. One-hour positive pressure ventilation after a successful spontaneous breathing trial: a multicenter feasibility randomized clinical trial. Crit Care Sci. 2025;37:e20250361.

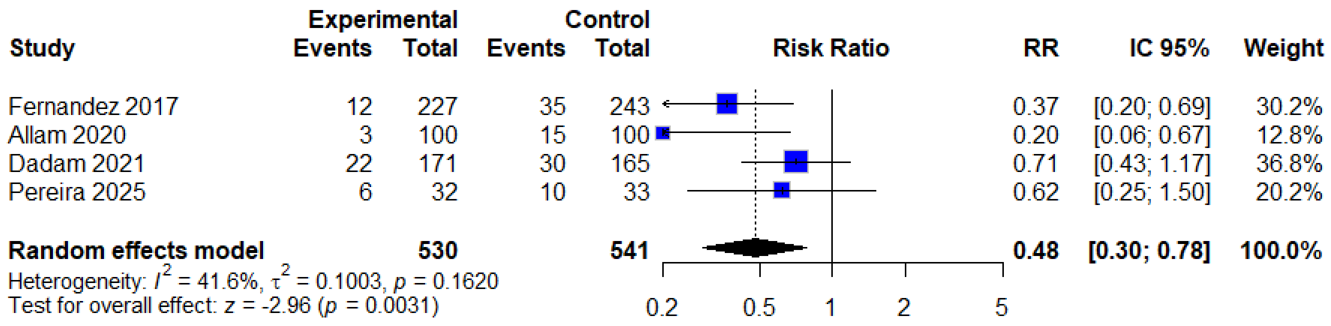


**Figure 1S -** Sensitivity analysis with pooled risk ratio of reintubation within 48 hours of randomization in patients treated with 1 hour of mechanical ventilation (Experimental Group) and immediate extubation (Control Group), using the DerSimonian-Laird method.

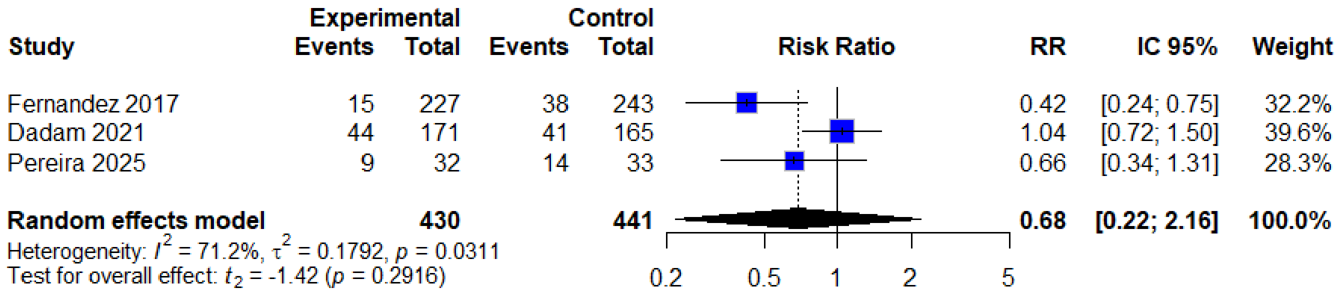


**Figure 2S -** Pooled risk ratio of extubation failure, defined as reintubation or death within 7 days after extubating, according to WIND classification in patients treated with 1 hour of mechanical ventilation (Experimental Group) and immediate extubation (Control Group) based on the Hartung-Knapp-Sidik-Jonkman random-effects model.

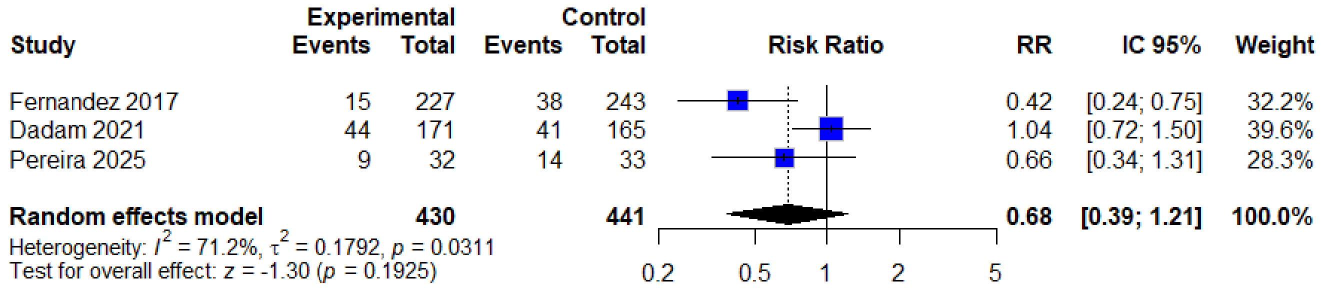


**Figure 3S -** Sensitivity analysis with pooled risk ratio of extubation failure, defined as reintubation or death within 7 days after extubating, according to WIND classification in patients treated with 1 hour of mechanical ventilation (Experimental Group) and immediate extubation (Control Group) based on the DerSimonian–Laird random-effects model.

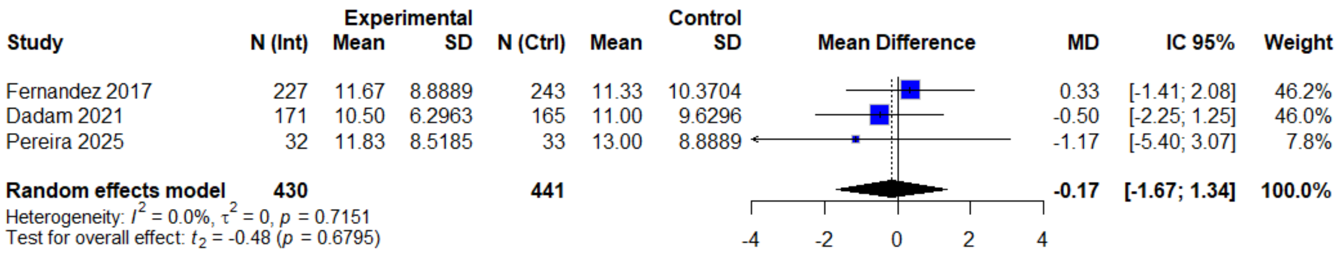


**Figure 4S -** Forest plot for the mean difference in intensive care unit length of stay in patients treated with 1 hour of mechanical ventilation (Experimental Group) and immediate extubation (Control Group) based on the Hartung-Knapp-Sidik-Jonkman random-effects model.

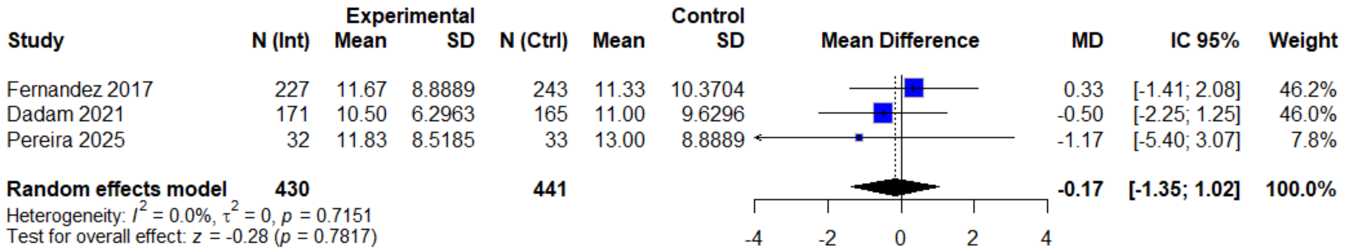


**Figure 5S -** Forest plot for the mean difference in intensive care unit length of stay in patients treated with 1 hour of mechanical ventilation (Experimental Group) and immediate extubation (Control Group) based on the DerSimonian–Laird random-effects model.

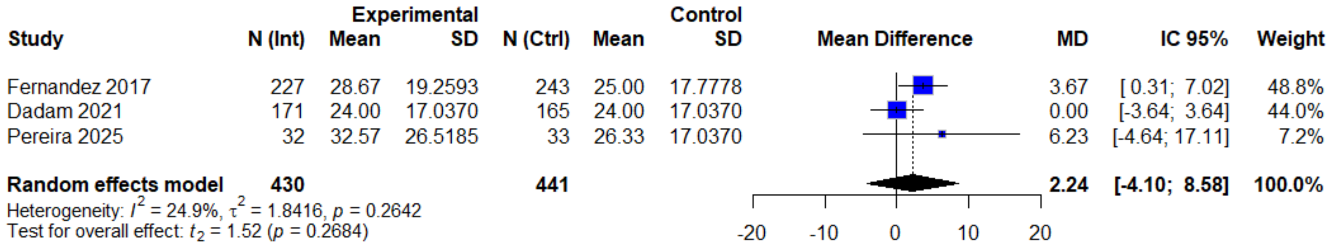


**Figure 6S -** Forest plot for the mean difference in hospital length of stay in patients treated with 1 hour of mechanical ventilation (Experimental Group) and immediate extubation (Control Group) based on the Hartung-Knapp-Sidik-Jonkman random-effects model.

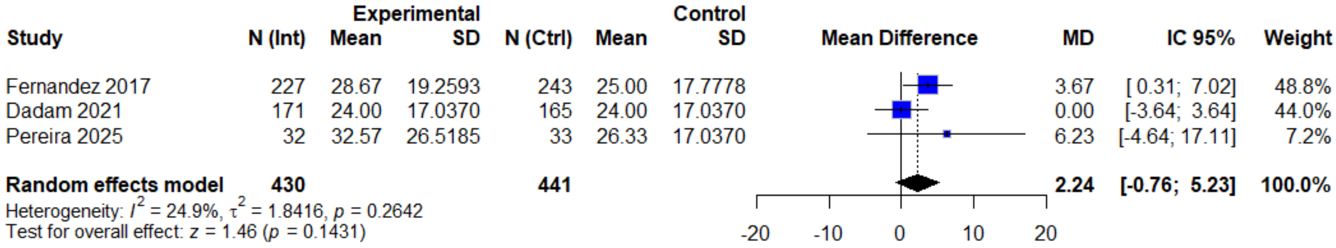


**Figure 7S -** Forest plot for the mean difference in hospital length of stay in patients treated with 1 hour of mechanical ventilation (Experimental Group) and immediate extubation (Control Group) based on the DerSimonian–Laird random-effects model.

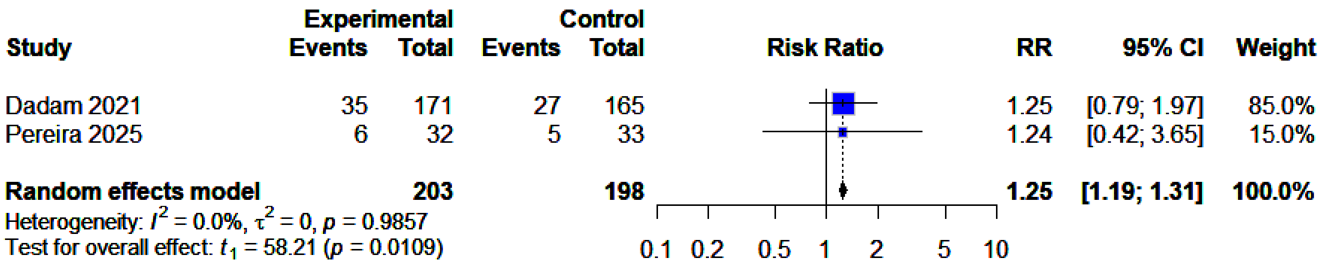


**Figure 8S -** Forest plot for in-hospital mortality in patients treated with 1 hour of mechanical ventilation (Experimental Group) and immediate extubation (Control Group) based on the random-effect model and Hartung–Knapp–Sidik–Jonkman method.

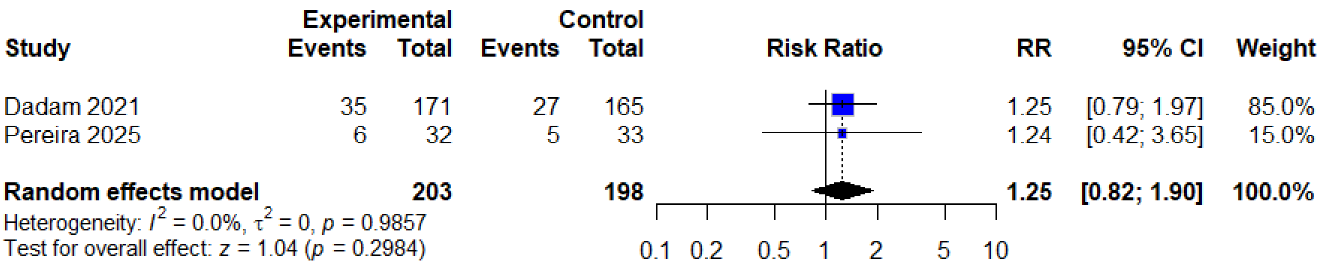


**Figure 9S -** Sensitivity analysis for in-hospital mortality in patients treated with 1 hour of mechanical ventilation (Experimental Group) and immediate extubation (Control Group) based on the DerSimonian-Laird random-effects model.

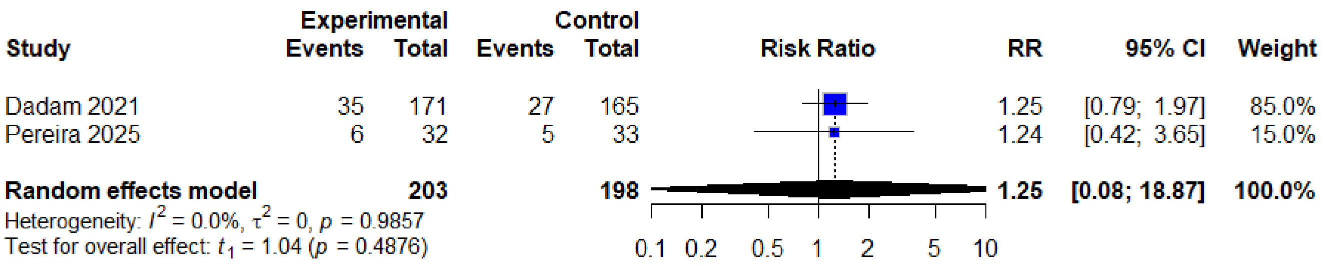


**Figure 10S -** Forest plot with ad hoc variance correction for in-hospital mortality in patients treated with 1 hour of mechanical ventilation (Experimental Group) and immediate extubation (Control Group) based on the random-effect model and modified Hartung–Knapp–Sidik–Jonkman method.

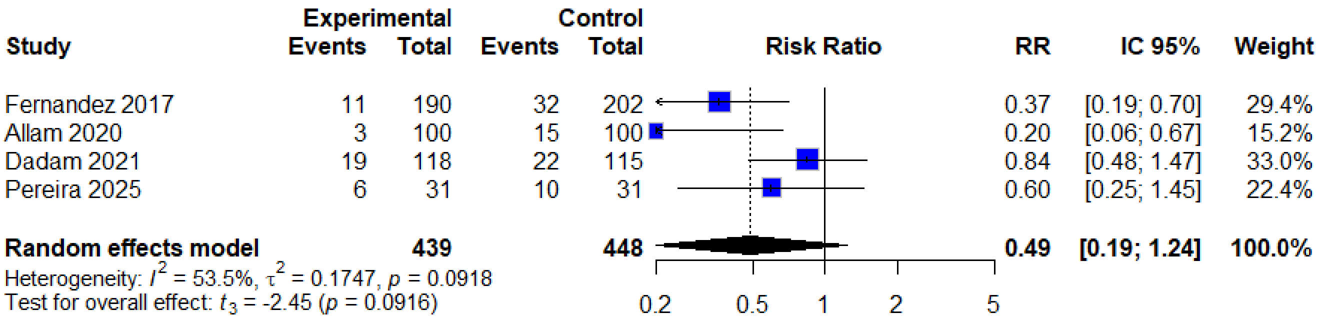


**Figure 11S -** Subgroup analysis of the pooled risk ratio of reintubation within 48 hours of randomization in high-risk of extubation failure patients treated with 1 hour of mechanical ventilation (Experimental Group) *versus* immediate extubation (Control Group).

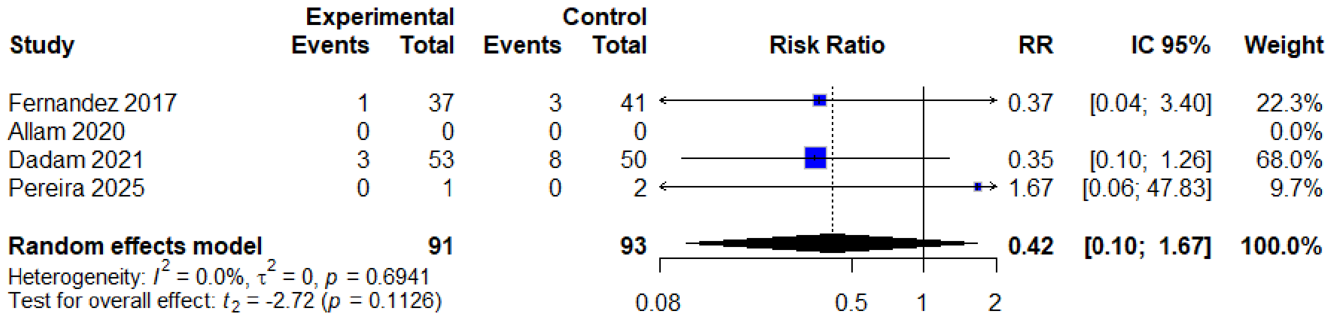


**Figure 12S -** Subgroup analysis of the pooled risk ratio of reintubation within 48 hours of randomization in low-risk of extubation failure patients treated with 1 hour of mechanical ventilation (Experimental Group) *versus* immediate extubation (Control Group).

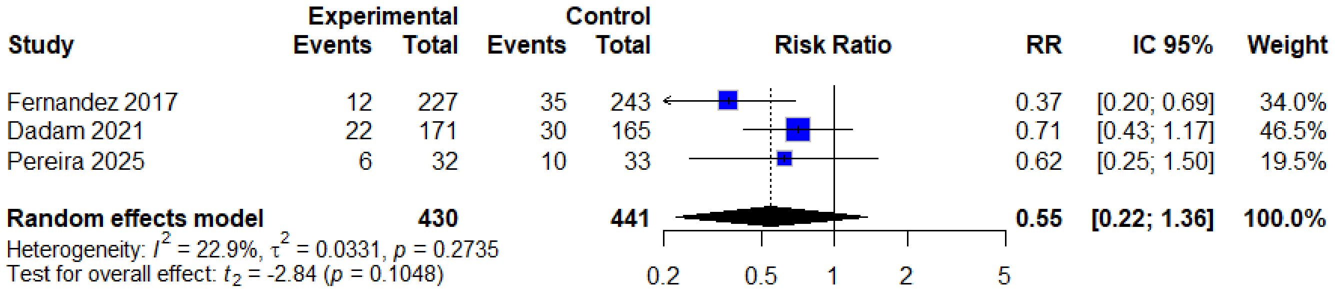


**Figure 13S -** Subgroup analysis of the pooled risk ratio of reintubation within 48 hours of randomization in studies with low risk of bias, comparing patients treated with 1 hour of mechanical ventilation (Experimental Group) *versus* immediate extubation (Control Group).
